# (*Z*)-4-[4-(Dimethyl­amino)benzyl­idene]-3-methyl­isoxazol-5(4*H*)-one

**DOI:** 10.1107/S160053680904714X

**Published:** 2009-11-14

**Authors:** Qingfang Cheng, Qi-fa Wang, Li-sha Liu, Jun-lei Zhang

**Affiliations:** a Huaihai Institute of Technology, Lianyungang 222005, People’s Republic of China

## Abstract

The title compound, C_13_H_14_N_2_O_2_, an isoxazol-5-one derivative, was synthesized by a one-pot, three-component condensation reaction of methyl acetoacetate, hydroxy­lamine hydro­chloride and 4-(dimethyl­amino)benzaldehyde. All the non-H atoms are co-planar [r.m.s deviation = 0.0039 Å], with a *Z* configuration about the C=C bond. The dihedral angle between the phenyl ring and the isoxazole ring is 2.58 (19)°.

## Related literature

For the biological activity of aryl­methyl­ene isoxazolone deriv­atives, see: Ishioka *et al.* (2002 ▶); Liu *et al.* (2005 ▶). For details of the synthesis of related compounds, see: Cocivera *et al.* (1976 ▶); Zhang *et al.* (2008 ▶); Villemin *et al.* (1993 ▶). For related structures, see: Kay *et al.* (2001 ▶); Wolf *et al.* (1995 ▶).
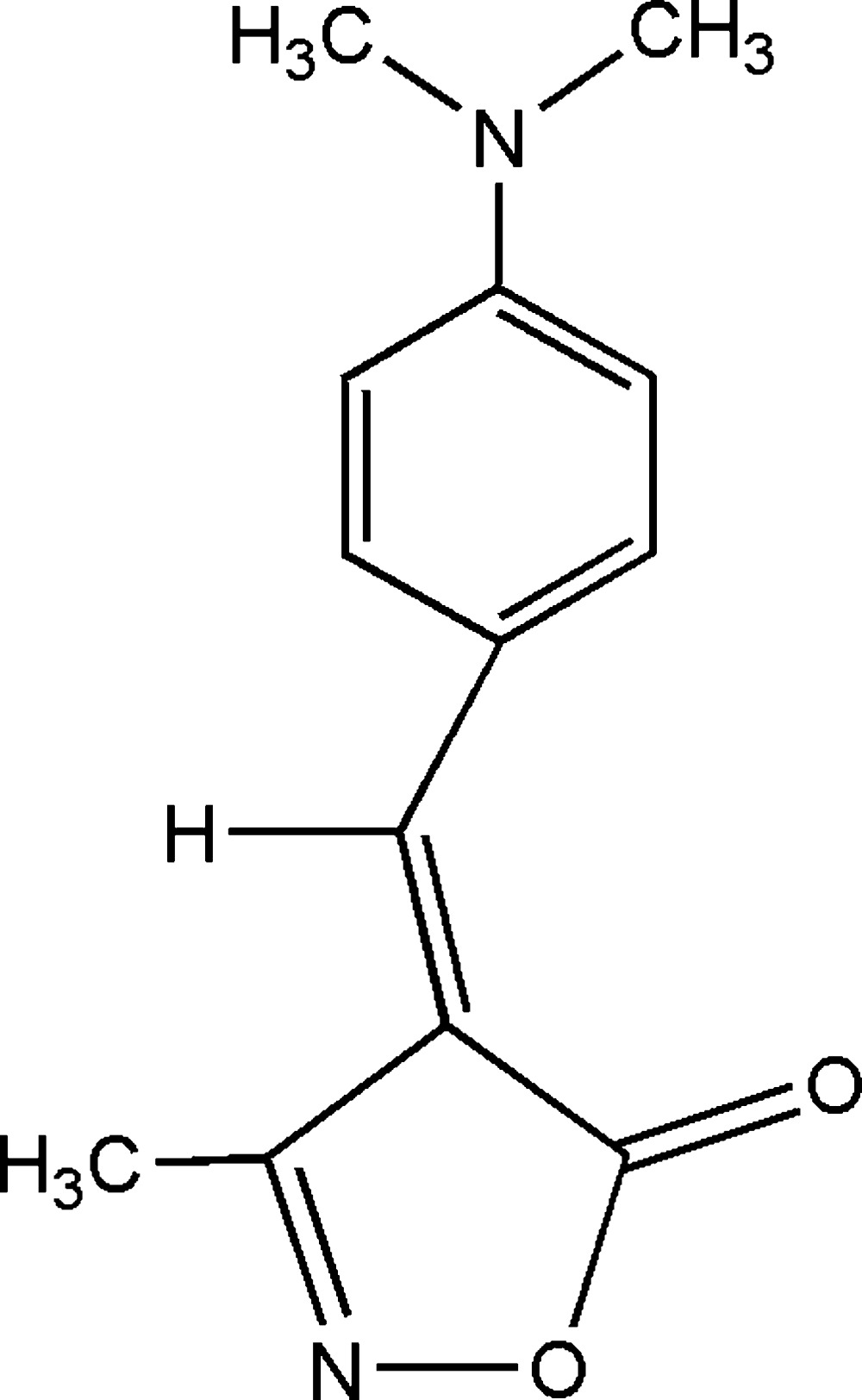


## Experimental

### 

#### Crystal data

C_13_H_14_N_2_O_2_
*M* *_r_* = 230.26
Triclinic, 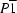

*a* = 6.4201 (10) Å
*b* = 7.8239 (12) Å
*c* = 12.1901 (15) Å
α = 100.272 (2)°
β = 97.319 (1)°
γ = 101.461 (2)°
*V* = 582.01 (15) Å^3^
*Z* = 2
Mo *K*α radiation
μ = 0.09 mm^−1^
*T* = 298 K
0.13 × 0.09 × 0.08 mm

#### Data collection

Bruker SMART CCD area-detector diffractometer
Absorption correction: multi-scan (*SADABS*; Sheldrick, 1996 ▶) *T*_min_ = 0.988, *T*_max_ = 0.993
2990 measured reflections
2020 independent reflections
943 reflections with *I* > 2σ(*I*)
*R*_int_ = 0.039

#### Refinement

*R*[*F* ^2^ > 2σ(*F* ^2^)] = 0.069
*wR*(*F* ^2^) = 0.178
*S* = 1.03
2020 reflections
157 parameters
H-atom parameters constrained
Δρ_max_ = 0.23 e Å^−3^
Δρ_min_ = −0.29 e Å^−3^

### 

Data collection: *SMART* (Bruker, 2007 ▶); cell refinement: *SAINT* (Bruker, 2007 ▶); data reduction: *SAINT*; program(s) used to solve structure: *SHELXS97* (Sheldrick, 2008 ▶); program(s) used to refine structure: *SHELXL97* (Sheldrick, 2008 ▶); molecular graphics: *SHELXTL* (Sheldrick, 2008 ▶); software used to prepare material for publication: *SHELXTL*.

## Supplementary Material

Crystal structure: contains datablocks I, global. DOI: 10.1107/S160053680904714X/su2156sup1.cif

Structure factors: contains datablocks I. DOI: 10.1107/S160053680904714X/su2156Isup2.hkl

Additional supplementary materials:  crystallographic information; 3D view; checkCIF report

## Figures and Tables

**Fig. 1.**
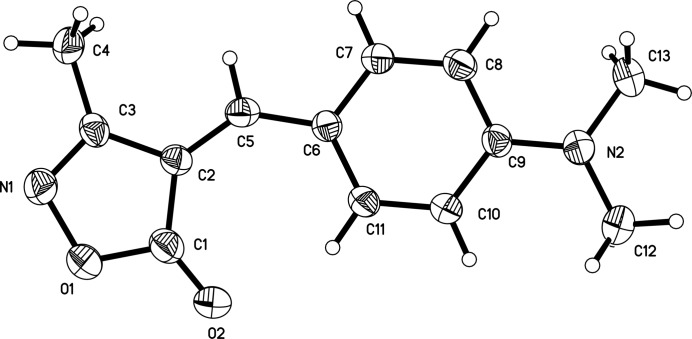
The molecular structure of the title compound with displacement ellipsoids drawn at the 30% probability level.

## supplementary crystallographic information

### Comment

Arylmethylene isoxazolone derivatives are effective anti-psychotics in the treatment of depression and schizophrenia. Studies on these compounds have mainly concentrated on their biological activities (Ishioka *et al.*, 2002; Liu *et al.*, 2005), and syntheses (Cocivera *et al.*, 1976; Zhang *et al.*, 2008; Villemin *et al.*, 1993). However, structural studies have rarely been reported (Kay *et al.*, 2001; Wolf *et al.*, 1995). As part of our investigations on arylmethylene isoxazolone derivatives, we report herein on the structure of the title compound. It was synthesized by a three component condensation reaction of methyl acetoacetate, hydroxylamine with 4-dimethylaminobenzaldehyde, in aqueous media under ultrasonic irradiation.

The molecular structure of the title compound is illustrated in Fig. 1, and geometrical parameters are given in the archived CIF. The bond lengths and angles agree well with those reported for the related compound 4-(N-(2,4,6-Tri-t-butylphenyl)iminomethylene)-3-t-butylisoxazol-5(4H)-one (Wolf *et al.*, 1995). The molecular structure adopts a planar conformation with *Z*-configuration about the C2═C5 double bond.

### Experimental

A mixture of methyl acetoacetate (4 mol), hydroxylamine hydrochloride (4 mmol), and pyridine(4 mmol) in distilled water(10 ml) was irradiated in the water bath of an ultrasonic cleaner for 10 min, then 4-dimethylaminobenzaldehyde(4 mmol) was slowly added to the mixture. The resulting mixture was irradiated in the water bath of an ultrasonic cleaner for 0.5 h. The solution was kept at r.t. overnight, giving a turbid solution. It was filtered to give a solid that was washed with cold water and ethanol. The crude product was recrystallized from ethanol to afford the title compound as a yellow solid. Single crystals, suitable for X-ray analysis, were obtained by slow evaporation of an aqueous ethanol (95%) solution at ambient temperature after 4 d. Elemental analysis, calculated for C_13_H_14_N_2_O_2_: C 67.81, H 6.13, N 12.17%; found: C 67.87, H 6.19, N 12.11%.

### Refinement

The H-atoms were included in calculated positions and allowed to ride on their parent atoms: C—H = 0.93–0.96 A °, with *U*_iso_(H) = 1.2U_eq_(C,*O*).

### Crystal data

**Table:**

C_13_H_14_N_2_O_2_	*Z* = 2
*M**_r_* = 230.26	*F*(000) = 244
Triclinic, *P*1	*D*_x_ = 1.314 Mg m^−^^3^
Hall symbol: -P 1	Mo *K*α radiation, λ = 0.71073 Å
*a* = 6.4201 (10) Å	Cell parameters from 607 reflections
*b* = 7.8239 (12) Å	θ = 2.9–25.1°
*c* = 12.1901 (15) Å	µ = 0.09 mm^−^^1^
α = 100.272 (2)°	*T* = 298 K
β = 97.319 (1)°	Needle, red
γ = 101.461 (2)°	0.13 × 0.09 × 0.08 mm
*V* = 582.01 (15) Å^3^	

### Data collection

**Table:**

Bruker SMART CCD area-detector diffractometer	2020 independent reflections
Radiation source: fine-focus sealed tube	943 reflections with *I* > 2σ(*I*)
graphite	*R*_int_ = 0.039
φ and ω scans	θ_max_ = 25.0°, θ_min_ = 2.9°
Absorption correction: multi-scan (*SADABS*; Sheldrick, 1996)	*h* = −7→7
*T*_min_ = 0.988, *T*_max_ = 0.993	*k* = −9→6
2990 measured reflections	*l* = −13→14

### Refinement

**Table:**

Refinement on *F*^2^	Primary atom site location: structure-invariant direct methods
Least-squares matrix: full	Secondary atom site location: difference Fourier map
*R*[*F*^2^ > 2σ(*F*^2^)] = 0.069	Hydrogen site location: inferred from neighbouring sites
*wR*(*F*^2^) = 0.178	H-atom parameters constrained
*S* = 1.03	*w* = 1/[σ^2^(*F*_o_^2^) + (0.069*P*)^2^ + ] where *P* = (*F*_o_^2^ + 2*F*_c_^2^)/3
2020 reflections	(Δ/σ)_max_ < 0.001
157 parameters	Δρ_max_ = 0.23 e Å^−^^3^
0 restraints	Δρ_min_ = −0.29 e Å^−^^3^

### Special details

**Table:**

Geometry. All e.s.d.'s (except the e.s.d. in the dihedral angle between two l.s. planes) are estimated using the full covariance matrix. The cell e.s.d.'s are taken into account individually in the estimation of e.s.d.'s in distances, angles and torsion angles; correlations between e.s.d.'s in cell parameters are only used when they are defined by crystal symmetry. An approximate (isotropic) treatment of cell e.s.d.'s is used for estimating e.s.d.'s involving l.s. planes.
Refinement. Refinement of *F*^2^ against ALL reflections. The weighted *R*-factor *wR* and goodness of fit *S* are based on *F*^2^, conventional *R*-factors *R* are based on *F*, with *F* set to zero for negative *F*^2^. The threshold expression of *F*^2^ > σ(*F*^2^) is used only for calculating *R*-factors(gt) *etc*. and is not relevant to the choice of reflections for refinement. *R*-factors based on *F*^2^ are statistically about twice as large as those based on *F*, and *R*- factors based on ALL data will be even larger.

### Fractional atomic coordinates and isotropic or equivalent isotropic displacement parameters (Å^2^)

**Table:**

	*x*	*y*	*z*	*U*_iso_*/*U*_eq_	
N1	0.7351 (5)	0.6599 (4)	0.8929 (3)	0.0671 (10)	
N2	0.3808 (5)	0.8488 (4)	0.1943 (3)	0.0604 (9)	
O1	0.9130 (4)	0.7385 (4)	0.8421 (2)	0.0746 (9)	
O2	0.9643 (4)	0.8104 (4)	0.6764 (2)	0.0797 (9)	
C1	0.8347 (6)	0.7524 (5)	0.7333 (4)	0.0626 (11)	
C2	0.6035 (5)	0.6855 (5)	0.7140 (3)	0.0492 (9)	
C3	0.5624 (5)	0.6321 (4)	0.8187 (3)	0.0503 (9)	
C4	0.3517 (5)	0.5490 (5)	0.8451 (3)	0.0634 (11)	
H4A	0.3722	0.5237	0.9195	0.095*	
H4B	0.2895	0.4400	0.7907	0.095*	
H4C	0.2568	0.6293	0.8423	0.095*	
C5	0.4464 (5)	0.6758 (4)	0.6248 (3)	0.0506 (9)	
H5	0.3094	0.6266	0.6373	0.061*	
C6	0.4428 (5)	0.7234 (4)	0.5171 (3)	0.0458 (9)	
C7	0.2412 (5)	0.6921 (5)	0.4469 (3)	0.0546 (10)	
H7	0.1181	0.6420	0.4730	0.066*	
C8	0.2192 (5)	0.7323 (5)	0.3417 (3)	0.0563 (10)	
H8	0.0829	0.7086	0.2980	0.068*	
C9	0.4013 (5)	0.8091 (4)	0.2993 (3)	0.0496 (9)	
C10	0.6035 (5)	0.8435 (5)	0.3700 (3)	0.0526 (10)	
H10	0.7264	0.8965	0.3448	0.063*	
C11	0.6244 (5)	0.8012 (4)	0.4750 (3)	0.0507 (9)	
H11	0.7605	0.8244	0.5187	0.061*	
C12	0.5668 (6)	0.9262 (5)	0.1481 (3)	0.0691 (12)	
H12A	0.6721	1.0058	0.2079	0.104*	
H12B	0.5224	0.9911	0.0929	0.104*	
H12C	0.6287	0.8329	0.1129	0.104*	
C13	0.1725 (6)	0.8003 (6)	0.1198 (3)	0.0751 (12)	
H13A	0.1191	0.6734	0.1050	0.113*	
H13B	0.1886	0.8376	0.0499	0.113*	
H13C	0.0725	0.8581	0.1555	0.113*	

### Atomic displacement parameters (Å^2^)

**Table:**

	*U*^11^	*U*^22^	*U*^33^	*U*^12^	*U*^13^	*U*^23^
N1	0.0601 (19)	0.091 (3)	0.058 (2)	0.0251 (18)	0.0110 (18)	0.027 (2)
N2	0.057 (2)	0.066 (2)	0.057 (2)	0.0083 (16)	0.0014 (17)	0.0214 (18)
O1	0.0534 (16)	0.105 (2)	0.0670 (19)	0.0177 (15)	0.0012 (14)	0.0282 (17)
O2	0.0470 (15)	0.116 (2)	0.075 (2)	0.0066 (15)	0.0082 (14)	0.0298 (18)
C1	0.053 (2)	0.076 (3)	0.058 (3)	0.018 (2)	0.003 (2)	0.012 (2)
C2	0.0429 (19)	0.056 (2)	0.050 (2)	0.0151 (16)	0.0067 (17)	0.0117 (19)
C3	0.051 (2)	0.059 (2)	0.045 (2)	0.0233 (18)	0.0035 (18)	0.0117 (19)
C4	0.067 (2)	0.077 (3)	0.055 (3)	0.020 (2)	0.013 (2)	0.027 (2)
C5	0.0423 (18)	0.049 (2)	0.063 (3)	0.0112 (16)	0.0113 (18)	0.016 (2)
C6	0.046 (2)	0.046 (2)	0.048 (2)	0.0124 (16)	0.0067 (17)	0.0134 (18)
C7	0.044 (2)	0.061 (3)	0.060 (3)	0.0079 (17)	0.0069 (18)	0.021 (2)
C8	0.046 (2)	0.058 (2)	0.062 (3)	0.0066 (18)	0.0001 (18)	0.016 (2)
C9	0.051 (2)	0.047 (2)	0.052 (2)	0.0118 (17)	0.0030 (18)	0.0161 (19)
C10	0.0437 (19)	0.059 (2)	0.055 (2)	0.0097 (17)	0.0045 (18)	0.016 (2)
C11	0.0435 (19)	0.055 (2)	0.054 (2)	0.0119 (17)	0.0021 (17)	0.0155 (19)
C12	0.075 (3)	0.077 (3)	0.060 (3)	0.016 (2)	0.015 (2)	0.027 (2)
C13	0.071 (3)	0.096 (3)	0.059 (3)	0.018 (2)	−0.002 (2)	0.029 (2)

### Geometric parameters (Å, °)

**Table:**

N1—C3	1.294 (4)	C6—C11	1.405 (4)
N1—O1	1.451 (3)	C6—C7	1.409 (5)
N2—C9	1.367 (4)	C7—C8	1.371 (5)
N2—C12	1.455 (4)	C7—H7	0.9300
N2—C13	1.457 (4)	C8—C9	1.407 (4)
O1—C1	1.388 (4)	C8—H8	0.9300
O2—C1	1.220 (4)	C9—C10	1.412 (5)
C1—C2	1.446 (5)	C10—C11	1.375 (4)
C2—C5	1.365 (5)	C10—H10	0.9300
C2—C3	1.451 (4)	C11—H11	0.9300
C3—C4	1.481 (4)	C12—H12A	0.9600
C4—H4A	0.9600	C12—H12B	0.9600
C4—H4B	0.9600	C12—H12C	0.9600
C4—H4C	0.9600	C13—H13A	0.9600
C5—C6	1.426 (4)	C13—H13B	0.9600
C5—H5	0.9300	C13—H13C	0.9600
			
C3—N1—O1	106.5 (3)	C8—C7—H7	118.7
C9—N2—C12	121.9 (3)	C6—C7—H7	118.7
C9—N2—C13	120.9 (3)	C7—C8—C9	120.4 (3)
C12—N2—C13	117.0 (3)	C7—C8—H8	119.8
C1—O1—N1	109.2 (3)	C9—C8—H8	119.8
O2—C1—O1	118.0 (3)	N2—C9—C8	120.8 (3)
O2—C1—C2	134.7 (4)	N2—C9—C10	122.0 (3)
O1—C1—C2	107.3 (3)	C8—C9—C10	117.2 (3)
C5—C2—C1	132.2 (3)	C11—C10—C9	122.0 (3)
C5—C2—C3	124.2 (3)	C11—C10—H10	119.0
C1—C2—C3	103.6 (3)	C9—C10—H10	119.0
N1—C3—C2	113.3 (3)	C10—C11—C6	120.9 (3)
N1—C3—C4	119.5 (3)	C10—C11—H11	119.5
C2—C3—C4	127.1 (3)	C6—C11—H11	119.5
C3—C4—H4A	109.5	N2—C12—H12A	109.5
C3—C4—H4B	109.5	N2—C12—H12B	109.5
H4A—C4—H4B	109.5	H12A—C12—H12B	109.5
C3—C4—H4C	109.5	N2—C12—H12C	109.5
H4A—C4—H4C	109.5	H12A—C12—H12C	109.5
H4B—C4—H4C	109.5	H12B—C12—H12C	109.5
C2—C5—C6	135.0 (3)	N2—C13—H13A	109.5
C2—C5—H5	112.5	N2—C13—H13B	109.5
C6—C5—H5	112.5	H13A—C13—H13B	109.5
C11—C6—C7	116.8 (3)	N2—C13—H13C	109.5
C11—C6—C5	125.4 (3)	H13A—C13—H13C	109.5
C7—C6—C5	117.8 (3)	H13B—C13—H13C	109.5
C8—C7—C6	122.7 (3)		
			
C3—N1—O1—C1	−0.9 (4)	C2—C5—C6—C7	−179.7 (4)
N1—O1—C1—O2	−177.5 (3)	C11—C6—C7—C8	0.8 (5)
N1—O1—C1—C2	1.1 (4)	C5—C6—C7—C8	−179.8 (3)
O2—C1—C2—C5	−5.2 (8)	C6—C7—C8—C9	−0.4 (5)
O1—C1—C2—C5	176.5 (4)	C12—N2—C9—C8	−179.2 (3)
O2—C1—C2—C3	177.5 (4)	C13—N2—C9—C8	−4.7 (5)
O1—C1—C2—C3	−0.9 (4)	C12—N2—C9—C10	1.1 (5)
O1—N1—C3—C2	0.4 (4)	C13—N2—C9—C10	175.6 (3)
O1—N1—C3—C4	178.9 (3)	C7—C8—C9—N2	179.7 (3)
C5—C2—C3—N1	−177.3 (3)	C7—C8—C9—C10	−0.6 (5)
C1—C2—C3—N1	0.3 (4)	N2—C9—C10—C11	−179.0 (3)
C5—C2—C3—C4	4.2 (6)	C8—C9—C10—C11	1.3 (5)
C1—C2—C3—C4	−178.2 (3)	C9—C10—C11—C6	−0.9 (5)
C1—C2—C5—C6	0.8 (7)	C7—C6—C11—C10	−0.1 (5)
C3—C2—C5—C6	177.6 (3)	C5—C6—C11—C10	−179.5 (3)
C2—C5—C6—C11	−0.4 (6)		
